# Genetically Predicted 1400 Blood Metabolites in Relation to Risk of Prostate Cancer: A Mendelian Randomization Study

**DOI:** 10.1002/agm2.70016

**Published:** 2025-06-11

**Authors:** Xiaojin Lu, Yongming Chen, Yuxiao Jiang, Jiaxin Ning, Shengjie Liu, Zhengtong Lv, Miao Wang, Huiming Hou, Ming Liu

**Affiliations:** ^1^ Medical School of University of Chinese Academy of Sciences Beijing China; ^2^ Beijing Hospital, National Center of Gerontology, Institute of Geriatric Medicine Chinese Academy of Medical Sciences & Peking Union Medical College Beijing China; ^3^ Department of Urology, Beijing Hospital National Center of Gerontology Beijing China

**Keywords:** blood metabolites, Mendelian randomization analysis, meta‐analysis

## Abstract

**Objectives:**

Metabolic dysregulation is common in cancer, yet evidence linking circulating metabolites to causal relationships in prostate cancer (PCa) is lacking. We performed a Mendelian randomization analysis utilizing 1400 blood metabolites to evaluate their roles in PCa.

**Methods:**

Exposure data from genome‐wide association studies (GWAS) was extracted from metabolite level GWAS involving 462,933 individuals of European descent. GWAS data for PCa were obtained from the UK Biobank (UKB) database (79,148 cases, 61,106 controls) for a two‐sample Mendelian randomization (MR) preliminary analysis, where we investigated potential causal relationships between 1400 metabolites and PCa. Inverse variance weighting (IVW) was the primary method for causal analysis, with MR‐Egger and weighted median as supplementary analyses to enhance robustness. Sensitivity analyses including Cochran *Q* test, MR‐Egger intercept test, MR‐PRESSO, and leave‐one‐out analysis were employed to evaluate the robustness of MR results. For significant associations, an additional independent PCa dataset was utilized for validation analysis and meta‐analysis.

**Results:**

Our findings revealed significant associations between two metabolites and prostate cancer: Cysteinylglycine disulfide levels (OR: 0.999, 95% CI: 0.998–0.999, *p* = 0.004). Validation analyses showed a similar trend, and sensitivity analyses supported the robustness of MR estimates.

**Conclusions:**

Our results suggest that Cysteinylglycine disulfide levels may have a causal relationship with increased PCa risk.

## Graphical abstract

This study employed Mendelian Randomization to assess 1,400 blood metabolites and found a significant inverse association between the glycine‐to‐serine ratio and prostate cancer risk, suggesting a potential causal relationship.

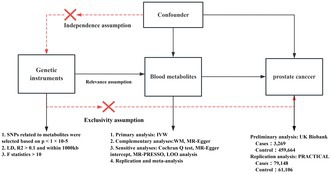

## 1 Introduction

Prostate cancer (PCa) is the most common solid organ tumor among men in developed countries and the second most prevalent globally [1]. In Western countries, it is the most common cancer among men aged 50 and older, with a mortality rate of 20% [2]. Worldwide, there are projected 1.4 million new cases and 375,000 deaths, making PCa the second most common malignancy and the fifth leading cause of cancer mortality in men, imposing a significant health burden on men in the US and globally [3].

Many PCa cases are currently detected through prostate‐specific antigen (PSA) screening, but the clinical significance of cases detected this way is limited. Research has shown that more aggressive, potentially lethal prostate cancers have different etiologies compared to indolent forms. Given this situation, enhancing prevention and screening for PCa is a critical priority. Despite significant advances in the past decade, early detection and treatment of PCa remain unsatisfactory.

Accumulating evidence from basic research indicates that cancer cells continuously adapt to the dynamic metabolic microenvironment by altering nutrient utilization during malignant transformation. Therefore, metabolic changes and pathway disruptions play significant roles in the development and progression of PCa; recently, the emergence of metabolomics as a component of systems biology has provided a novel approach to studying disease mechanisms. Specifically, metabolomics can deepen our understanding of the biological mechanisms of diseases by identifying modified metabolites or metabolic pathways. However, large‐scale human data are needed to clarify these associations [4, 5]. Increasingly, epidemiological studies are leveraging metabolomics to investigate the etiology of PCa and identify biomarkers for early detection or progression [4].

Thus, exploring metabolites associated with PCa not only aids in early screening and prevention but also contributes to understanding the biological mechanisms underlying PCa for therapeutic purposes. One way to assess the role of metabolites in disease outcomes is through human genetics. In recent years, several genome‐wide association studies (GWAS) have achieved substantial progress in revealing the genetic determinants of the human metabolome [6, 7, 8]. Many metabolite levels exhibit high heritability [9, 10]. Mendelian randomization (MR) analysis is an emerging method that uses genetic variation as proxies for exposure to assess causal relationships between exposures (in this case, metabolites) and disease outcomes [11]. MR assesses the causal impact of the genetic proxy exposure on outcomes by selecting single nucleotide polymorphisms (SNPs) associated with the exposure as instrumental variables (IVs) [12]. The establishment of genotypes occurs before disease onset and is essentially unaffected by disease progression, enabling effective assessment of the causal relationship between metabolites and PCa [13, 14].

Due to the lack of understanding of the causal relationships between blood metabolites and PCa, further research is warranted in this field. In this work, we conducted an MR analysis using summarized data from GWAS to comprehensively explore the causal impact of 1400 blood metabolites on PCa. This study aims to reveal the etiology associated with metabolite‐related PCa and to gain insights into its biological processes.

## 2 Methods

### 2.1 Study Design

**FIGURE 1:**
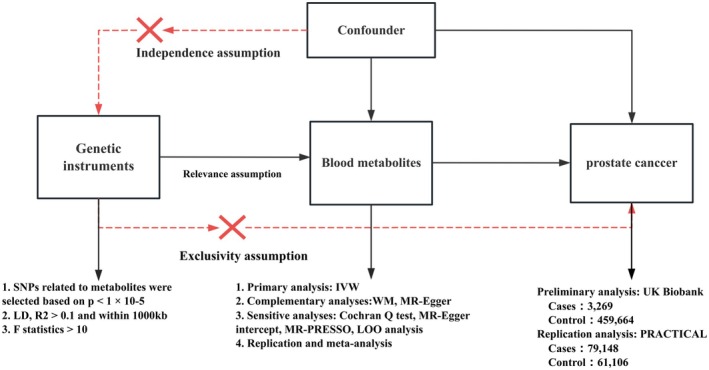
Study design.

Effective MR studies must satisfy three assumptions: (1) the IV is closely related to the exposure of interest; (2) the IV is independent of confounding factors; (3) the IV is associated with the outcome only through the exposure [15]. All MR analyses in this study were conducted using the Two Sample MR, MR‐PRESSO, and Radial MR packages in R software (version 4.2.1). An overview of the study is depicted in Figure 1. The MR study design referenced that of Cai et al. [16].

To comprehensively assess the robustness of candidate metabolites identified based on the above criteria, we repeated inverse variance weighting (IVW) analysis across three PCa cohorts (one preliminary analysis and one validation analyses). Through meta‐analysis of the results from the two MR analyses, we ultimately identified blood metabolites causally related to PCa. Meta‐analysis was based on a fixed‐effects IVW model, utilizing Review Manager software (version 5.4). Sensitivity analyses included Cochran *Q* tests, leave‐one‐out analyses, funnel plots, and MR‐Egger intercept analyses. If heterogeneity or pleiotropy was present, the MR‐PRESSO method was used to detect outliers. Subsequently, after removing outliers and calculating causal effects, multiplicative random effects IVW was performed to assess heterogeneity.

### 2.2 Data Source for Blood Metabolome and Prostate Cancer

For preliminary analysis of PCa data, publicly available summary statistics were obtained from the UK Biobank (http://www.nealelab.is/uk‐biobank/) involving individuals of European ancestry diagnosed with PCa (total *n* = 462,933; ncase = 3269, ncontrol = 459,664; Phenotype code: 20001#1044). Validation analysis data were accessed from the Prostate Cancer Association Group to Investigate Cancer Associated Alterations in the Genome (PRACTICAL) consortium, including 79,194 cases and 61,112 controls [17].

The GWAS summary statistics for 1400 metabolites were stored in the GWAS catalog (https://www.ebi.ac.uk/gwas/). Accession numbers for European GWASs: GCST90199621–90201020. These statistics encompass a total of 1400 metabolites. Summary statistics of genetic variants associated with the human metabolome were derived from three large‐scale GWAS involving approximately 8000 individuals of European ancestry (Table 1) [18]. Furthermore, all participants in this study were of European descent, ensuring that there was no overlap between exposure and outcome characteristics to minimize bias caused by confounding factors.

**TABLE 1:** Data source.

Data	Data source	Author	Population	Sample size	Cases	Controls
PCa	UK Biobank	Ben Elsworth et al.	European	462,933	3269	459,664
PCa	PRACTICAL	Schumacher FR et al.	European	140,254	79,148	61,106
1400 plasma metabolitesa	GCST90199621‐GCST90201020	Yiheng Chen et al.	European	8299		

During sample quality control steps, individuals with ambiguous sex, high genotype missing rates (> 5%), excessive heterozygosity (± 4SD), and non‐Finnish ancestry were excluded. In the variant quality control steps, variants with high missingness (> 2%), low HWE *p*‐values (< 1e‐6), and low minor allele counts (MAC < 3) were excluded. Because our study was based on publicly available databases, no additional ethical approval or informed consent was required.

### 2.3 Selection Criteria for Genetic Variants

In selecting instrumental variables (IVs) to represent potential exposure–outcome associations, different thresholds were established based on changes in exposure. Initially, the 1400 metabolites were designated as exposures. In this case, SNPs with an association threshold of *p* < 1 × 10^−5^ were extracted, primarily for MR analyses, with a linkage disequilibrium parameter (*R* ^2^ = 0.001, window size = 10,000 kb) to obtain independent SNPs [19]. Given the moderate number of metabolite‐associated SNPs and to meet the minimum standards for MR studies, at least 10 qualifying instrumental variables (IVs) were required; thus, we adopted a more lenient threshold.

To further obtain independent SNPs, we pruned these instruments within a 10,000 kb window size to mitigate linkage disequilibrium (LD) at the *r* ^2^ < 0.001 threshold. Additionally, *R* ^2^ and *F* statistics for IVs were calculated to identify potential weak IV bias. SNPs with *F* < 10 were considered weak instruments and excluded to ensure that all SNPs in the exposure group had sufficient variation. Subsequently, exposure‐associated SNPs were separated from outcome‐associated SNPs. The MR Steiger filter was used to exclude SNPs with incorrect causal direction. During data harmonization, allele information for exposure and outcome SNPs was matched. Finally, at least two SNPs were retained for each metabolite for MR analyses. We also searched the PhenoScanner database(http://www.phenoscanner.medsci.org/) to identify the associations between IVs and potential confounders (e.g., age, body mass index) [20, 21, 22].

### 2.4 Statistical Analysis

We employed various methods to detect and adjust for heterogeneity and pleiotropy in estimating the associations of 1400 metabolites with prostate cancer. The analysis involved four steps: First, we clustered SNPs to obtain independent genetic instrumental variables. Second, we identified proxy SNPs for any missing SNPs. Third, we discarded SNPs significantly associated with the outcome. Fourth, ambiguous and palindromic SNPs were removed. Subsequently, we conducted the MR analysis. Specifically, inverse variance weighting (IVW) estimates were used as the primary MR effect estimates, reported as odds ratios (OR) and 95% confidence intervals (95% CI) [23]. Additionally, we employed three other methods to estimate causal effects: weighted median, weighted mode, and MR‐Egger regression [24]. These three methods are considered the most scientifically robust and commonly used for providing reliable analyses in Mendelian randomization studies.

Sensitivity analyses are essential for evaluating potential biases in Mendelian randomization studies. They include two considerations: heterogeneity testing and pleiotropy testing. A Cochran *Q* test yielding *p* < 0.05 indicates heterogeneity in the results. The MR‐PRESSO method [25] can detect and adjust for horizontal pleiotropy by removing outliers. To assess the robustness of the results [25], we conducted leave‐one‐out (LOO) analyses, sequentially excluding each SNP to determine if individual SNPs had a substantial impact on the MR estimates [26].

Among the 1091 plasma metabolites tested, 850 had known identities within eight super pathways (lipids, amino acids, xenobiotics, nucleotides, cofactors and vitamins, carbohydrates, peptides, and energy). The remaining 241 were classified as unknown or “partially” characterized molecules, with specific classifications provided in Table S1, which also includes metabolite ratios categorized into 10 classes [18]. To achieve a stricter interpretation of causality, we applied the Bonferroni method to set significance thresholds based on metabolite classification levels (5.6 × 10–3 [0.05/10]) [27]. *p* values achieving nominal significance (*p* < 0.005) were considered to indicate potential causal effects. The “TwoSampleMR” and “MR‐PRESSO” packages were utilized for analyses in R (version 4.2.2).

In summary, we rigorously screened blood metabolites with potential causal effects on prostate cancer through multiple criteria: (1) significant preliminary analysis *p*‐values (IVW *p* < 0.005), (2) consistent direction and magnitude across three MR methods, (3) absence of heterogeneity or horizontal pleiotropy in MR results, and (4) no severe interference of MR estimates by any single SNP.

## 3 Results

### 3.1 Preliminary Analysis

In the preliminary analysis, after strict quality control of instrumental variables (IVs), the MR study ultimately captured 1401 metabolites. The filtered IVs contained between 11 and 69 SNPs (with the genetic proxy for 3‐hydroxylaurate levels comprising 2 SNPs; 1‐stearoyl‐GPE (18:0) levels generating the most genetic proxies: 28 SNPs). All F‐statistics for SNPs associated with metabolites were greater than 10, indicating the strong power of the IVs. Detailed data on the IVs are provided in Table S2.

**FIGURE 2:**
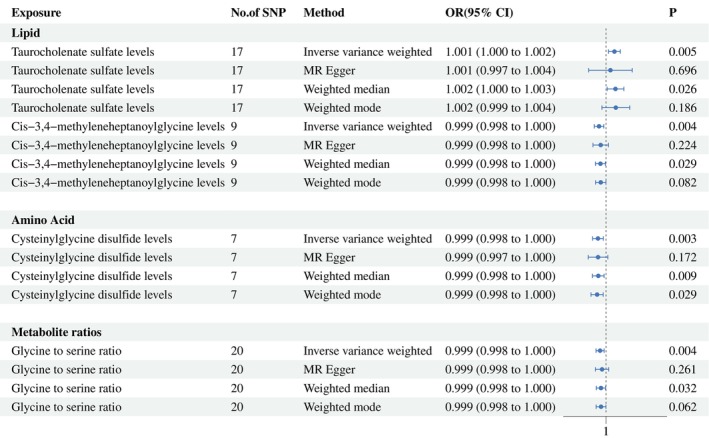
Forest plot for the causality of blood metabolites on colorectal cancer derived from inverse variance weighting, MR‐Egger regression, weighted median, and weighted mode analysis. CI, confidence interval; OR, odds ratio; SNPs, single nucleotide polymorphisms.

**FIGURE 3:**
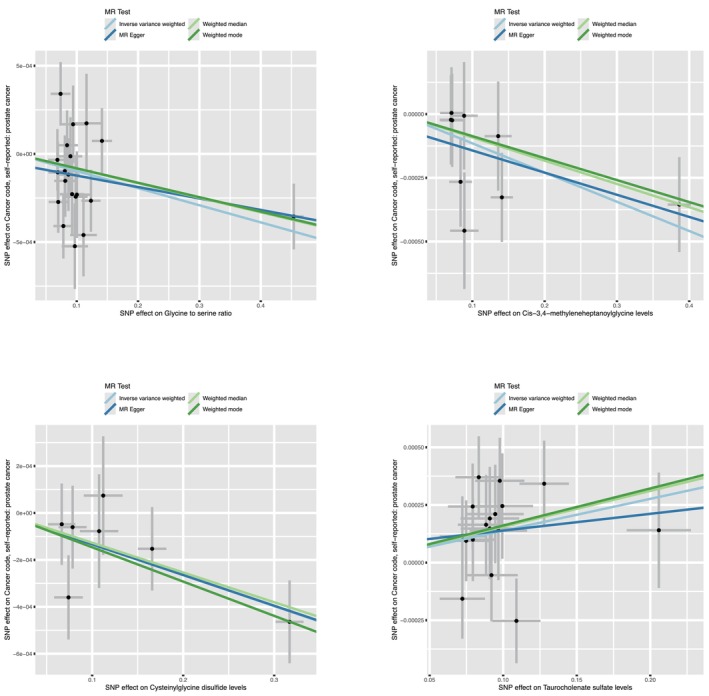
Scatterplot of significantly associated (IVW‐derived *p* < 0.05) and directionally consistent estimates. SNP, single nucleotide polymorphisms.

Using stringent criteria, we found consistent results across four MR models (IVW [*p* < 0.005], simultaneous IVW, MR‐Egger regression, and weighted median). The consistency of these four MR models enhances the reliability of serum metabolites in prostate cancer. Following complementary and sensitivity analyses, four plasma metabolites that met strict screening criteria were identified as candidate metabolites (Figures 2 and 3). To comprehensively assess any potential bias in our MR study, sensitivity analyses were conducted using complementary methods.

The summary results of the pleiotropy and heterogeneity tests are presented in Table 2, with Cochran *Q* tests (*p* > 0.05) and MR‐Egger intercept tests (*p* > 0.05) indicating no evidence of heterogeneity or pleiotropy. Additionally, MR‐PRESSO and Radial MR analyses also did not support the presence of heterozygous SNPs. Leave‐one‐out analyses were employed to verify the influence of each SNP on the overall causal estimates. When excluding a particular SNP, the remaining SNPs' meta‐effects did not cross zero, suggesting the results were consistent and reliable (Figure S1). The four identified blood metabolites were considered candidates for further analysis.

**TABLE 2:** Exposure and method results.

Exposure	Method	OR (95% CI)	*p*	Heterogeneity		Pleiotropy	
Taurocholenate sulfate levels	IVW	1–1.002	0.005	13.424	0.642		
Taurocholenate sulfate levels	ME	0.997–1.004	0.696	13.284	0.580	6.6E–05	0.714
Cis‐3,4‐methyleneheptanoylglycine levels	IVW	0.998–1	0.004	5.209	0.735		
Cis‐3,4‐methyleneheptanoylglycine levels	ME	0.998–1	0.224	4.922	0.670	−6E–05	0.609
Cysteinylglycine disulfide levels	IVW	0.998–1	0.003	3.299	0.770		
Cysteinylglycine disulfide levels	ME	0.997–1	0.172	3.299	0.654	−3E–06	0.984
Glycine to serine ratio	IVW	0.998–1	0.004	21.772	0.296		
Glycine to serine ratio	ME	0.998–1	0.261	21.143	0.272	−6E–05	0.474

Abbreviations: CI, confidence interval; IVW, inverse variance weighted; ME, MR‐Egger; OR, odds ratio.

### 3.2 Replication Analysis

**FIGURE 4:**
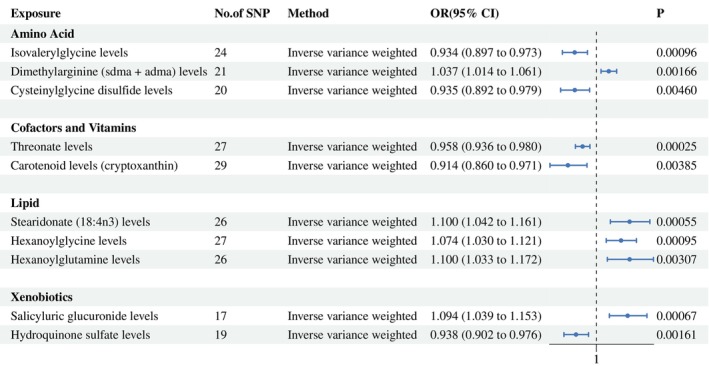
Forest plot for the causality of blood metabolites on colorectal cancer derived from inverse variance weighting. CI, confidence interval; OR, odds ratio; SNPs, single nucleotide polymorphisms.

To enhance the credibility of our estimates, we replicated the MR analysis using an independent GWAS dataset for prostate cancer (PCa). As expected, we applied strict criteria to screen the PRACTICAL cohort PCa GWAS data, resulting in 10 candidate plasma metabolites (Figure 4).

**FIGURE 5:**
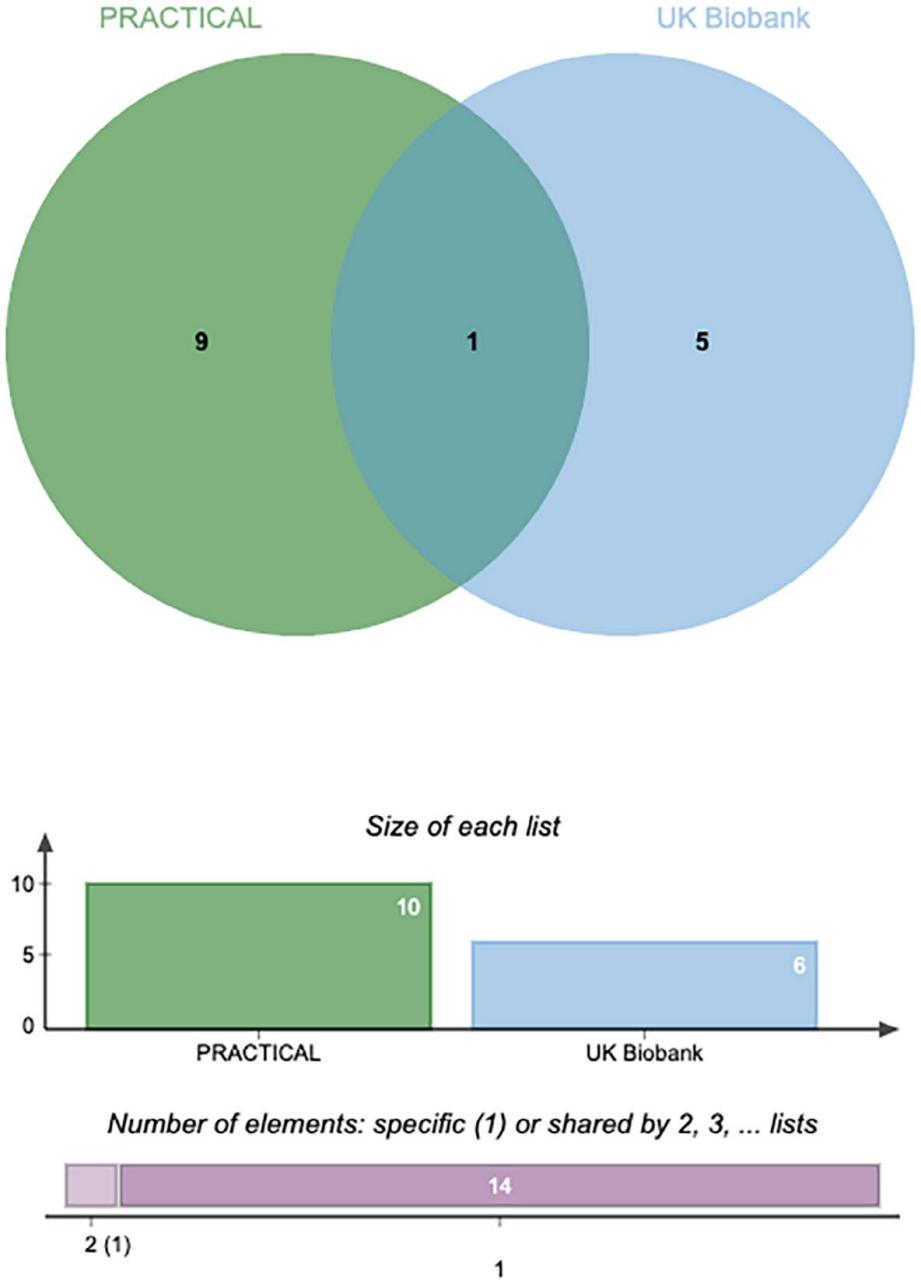
Venn diagram.

The intersection of plasma metabolites filtered from the two cohorts revealed only Cysteinylglycine disulfide levels. The replication analysis results further confirmed that Cysteinylglycine disulfide levels can influence PCa (Figure 5).

**FIGURE 6:**
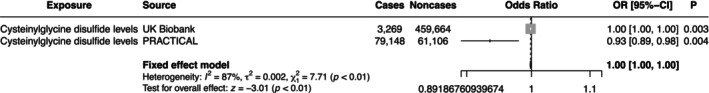
Meta‐analysis of significant association (IVW‐derived *p* < 0.05) between metabolites and prostate cancer. 95% CI, 95% confidence interval; OR, odds ratio.

Specifically, higher levels of Cysteinylglycine disulfide (UK Biobank cohort, OR: 0.999, 95% CI: 0.998–0.999, *p* = 0.004) were associated with reduced susceptibility to PCa, with consistent direction observed in both MR analyses (PRACTICAL cohort, OR: 0.934, 95% CI: 0.891–0.979, *p* = 0.004). The results of the meta‐analysis further confirmed that higher levels of Cysteinylglycine disulfide were associated with a reduced risk of prostate cancer (Figure 6).

## 4 Discussion

Observational studies have reported associations between metabolites and certain types of prostate cancer. In the current study, we integrated two large‐scale GWAS datasets and conducted two‐sample bidirectional MR analyses to explore the causal effects of 1400 blood metabolites on PCa. Our findings reveal complex interactions between metabolites and prostate cancer. In the preliminary MR analysis, we identified a total of 4 suggestive associations. Ultimately, we established that high levels of Cysteinylglycine disulfide are associated with a lower risk of prostate cancer, indicating a protective factor. We utilized available GWAS data and the two‐sample MR method to investigate the causal relationship between prostate cancer and metabolites. Extensive sensitivity analyses demonstrated that these associations are robust against the pleiotropy of the MR methods and tools used, with consistent results from MR‐PRESSO and leave‐one‐out analyses. The replication and meta‐analysis further enhanced the reliability of our results. To our knowledge, this is the first MR study to apply the most comprehensive blood metabolite GWAS data to explore causal relationships with prostate cancer.

In recent years, the high incidence and mortality rates of prostate cancer have imposed a significant burden worldwide, making the prevention of prostate cancer an urgent strategy. The advent of metabolomics technology has spurred interest in exploring the perceived value of metabolites in prostate cancer. Notably, blood metabolites provide a snapshot of biological mechanisms, capturing both endogenous and exogenous processes simultaneously [28, 29, 30]. Although previous studies have provided compelling evidence that metabolites are involved in the biological mechanisms of prostate cancer, their contribution to prevention has been limited due to unclear causal relationships. Therefore, we conducted this critical MR study to clarify the causal relationship between blood metabolites and prostate cancer, as well as the metabolic pathways involved, to provide guidance for prevention and treatment strategies.

Certain chemicals in hair dye may be carcinogenic to individuals with occupational exposure. A serum metabolomics study [31] suggested that the use of hair dye is strongly associated with a reduction in glutathione (GSH)‐related metabolites, particularly L‐cysteinylglycine disulfide (a marker of reduced glutathione production or excretion into extracellular compartments), which is consistent with the findings of our study. The reduction of L‐cysteinylglycine disulfide may increase the risk of prostate cancer. GSH serves as a key intracellular detoxifying agent in the metabolism of endogenous and exogenous compounds [32].

In exploring the mechanisms by which reduced cysteinylglycine may contribute to cancer development, cysteinylglycine, as a key intermediate in the GSH metabolic pathway, plays a vital role in maintaining the cell's antioxidant defense system. GSH neutralizes reactive oxygen species (ROS) and free radicals, which are closely associated with carcinogenesis [33]. A decrease in cysteinylglycine levels indicates a reduction in the cell's antioxidant capacity, thereby increasing oxidative stress and potentially leading to DNA damage and genetic mutations, which may promote cancer formation. Additionally, a reduction in cysteinylglycine may disrupt other critical cellular processes, such as DNA repair, cell cycle regulation, and apoptosis [34]. Dysregulation of these processes plays a crucial role in cancer initiation and progression. For example, a decrease in GSH may not only lead to reduced ROS clearance efficiency but also promote inflammatory responses, which are especially significant in certain cancer types [35]. By studying the metabolic processes of cysteinylglycine within cells, researchers have found that changes in its levels are closely associated with various cancers, and further exploration of its underlying mechanisms and therapeutic applications is of great significance. Overall, a reduction in cysteinylglycine may contribute to cancer development through increased oxidative stress, decreased antioxidant defenses, and dysregulation of critical metabolic pathways.

One of the major strengths of this study is the use of MR design in combination with the largest publicly available GWAS datasets. As alleles are randomly assigned and fixed at conception, MR analyses reduce the bias caused by confounding and reverse causality [36]. Another strength is that the sample was predominantly limited to individuals of European ancestry, which reduces bias due to population stratification. However, this limitation also restricts the generalizability of the results to this population group. Our findings offer the opportunity to test hypotheses for future similar studies, but given the dynamic nature of thiol‐disulfide exchange reactions and potential methodological artifacts interfering with its measurement, further research is required.

Nevertheless, larger sample sizes and more detailed GWAS data are needed to explore the preventive role of metabolites such as amino acids in prostate cancer. Another limitation is that both GWAS datasets come from European populations, and we do not know whether similar results would be obtained if extended to other racial groups.

## 5 Conclusion

Our study is based on MR research, which serves as a tool to test causal relationships. It cannot specify the exact magnitude or extent of causal effects, nor can it replace clinical trials [37]. However, based on the growing understanding of the influence of cysteinylglycine disulfide levels in blood metabolites on prostate cancer, these findings are certainly relevant to dietary recommendations and may have implications for cancer prevention and general public health guidance.

## Author Contributions

Xiaojin Lu conceptualized the study design, contributed to data extraction, data analysis, and drafted the manuscript. Yuxiao Jiang contributed to data extraction and analysis. Yongming Chen performed data checks. Ming Liu, Huiming Hou, Miao Wang, and Zhengtong Lv, reviewed the manuscript. All authors have read and approved the final manuscript for publication. All authors have read and approved the manuscript.

## Disclosure

We certify that the submission is an original work and this paper has not been published elsewhere in whole or in part. All authors have read and approved the content and agree to submit it for consideration for publication in your journal.

## Ethics Statement

The authors have nothing to report.

## Consent

The authors have nothing to report.

## Conflicts of Interest

The authors declare no conflicts of interest.

## Supporting information


Figure S1.

Table S1.

Table S2.

## Data Availability

All data analyzed in this study are included in the manuscript and [Link], [Link], [Link].
