# Supplementary figures and images for: Genetically Predicted 1400 Blood Metabolites in Relation to Risk of Prostate Cancer: A Mendelian Randomization Study
Source: Aging Med (Milton). 2025 Jun 11;8(3):249–57. doi: 10.1002/agm2.70016 (PMC12226420; doi:10.1002/agm2.70016)

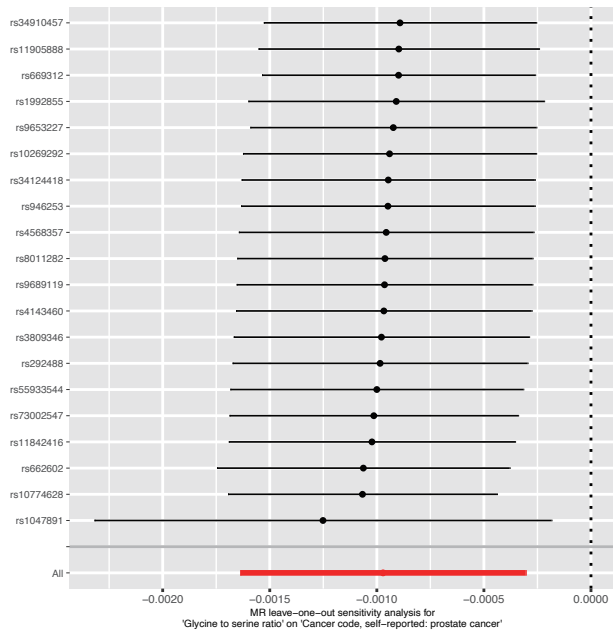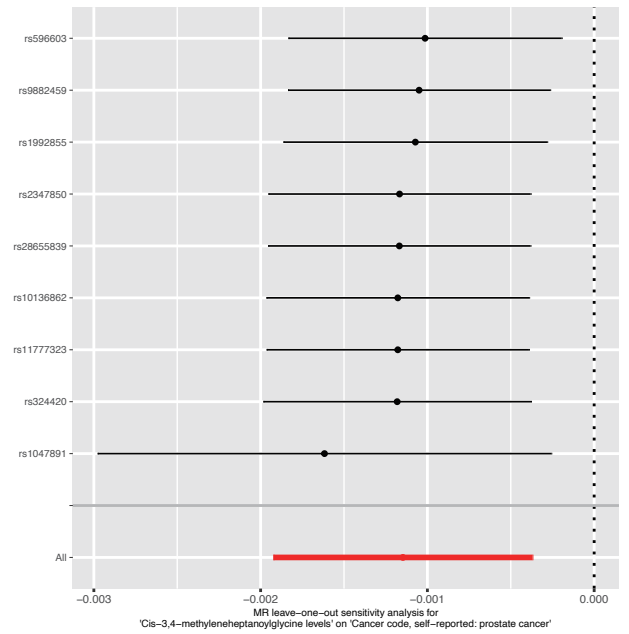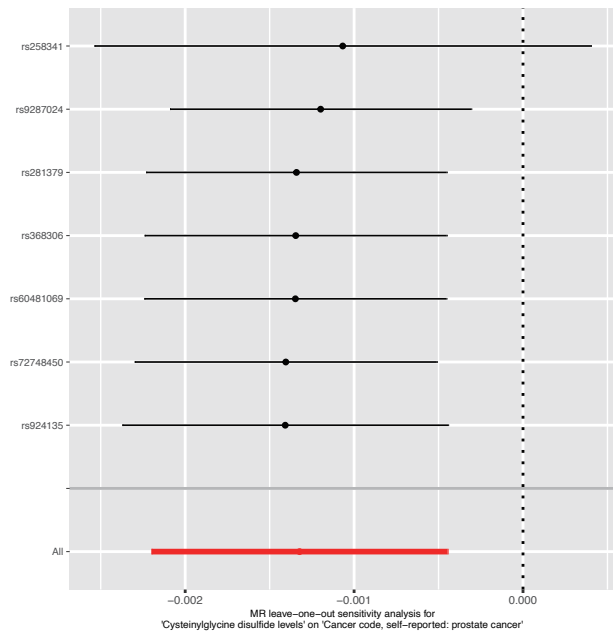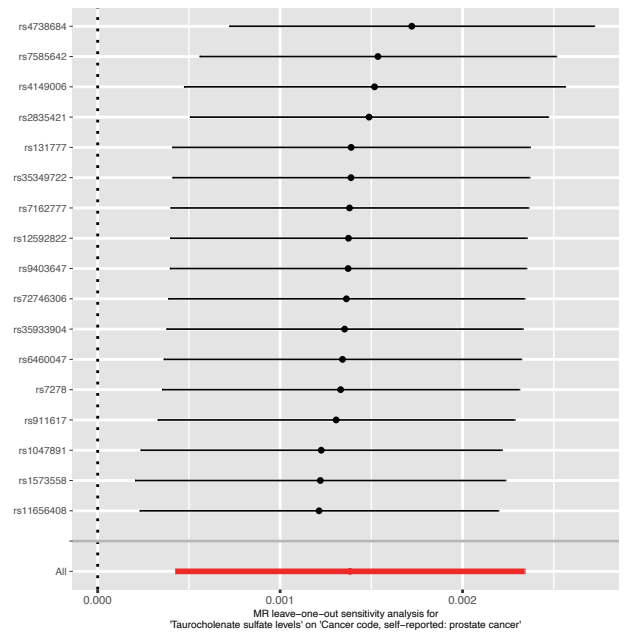

Supplement: Supplementary file 1 — Figure S1. [file AGM2-8--s003.pdf]
